# Allogeneic transplant procurement in the times of COVID-19: Quality report from the central European cryopreservation site

**DOI:** 10.1186/s12967-021-02810-9

**Published:** 2021-04-08

**Authors:** Eliza Wiercinska, Vera Schlipfenbacher, Gesine Bug, Peter Bader, Mareike Verbeek, Erhard Seifried, Halvard Bonig

**Affiliations:** 1 German Red Cross Blood Service Baden-Württemberg-Hesse, Institute Frankfurt, Frankfurt a.M., Germany; 2 Center for Internal Medicine, Department of Medicine II: Hematology, Oncology, Hemostaseology, Rheumatology and Infectious Diseases, Goethe University, Frankfurt, Germany; 3 Center for Child Health, Dept. of Oncology, Immunology and Stem Cell Transplantation, Goethe University, Frankfurt, Germany; 4 Clinic for Internal Medicine III, Hematology and Oncology, Klinikum Rechts Der Isar, Technical University Munich, Munich, Germany; 5 Institute for Transfusion Medicine and Immunohematology, Goethe University, Haus 76, Sandhofstraße 1, 60528 Frankfurt, Germany; 6 Dept. of Medicine/Division of Hematology, University of Washington, Seattle, WA USA

**Keywords:** Hematopoietic stem cell, Stem cell enumeration, CD34+ cell enumeration, Graft, Graft quality, Quality control, Engraftment, Stem cell dose, Post-thaw recovery, Transplant logistics

## Abstract

**Background:**

Because of limitations of transportation imposed by the COVID-19 pandemic, current recommendation calls for cryopreservation of allogeneic stem cell transplants before patient conditioning. A single cell therapy laboratory was selected to function as the central cryopreservation hub for all European registry donor transplants intended for the Australian-Pacific region. We examined properties of these transplants to ascertain how quality is maintained.

**Methods:**

We analyzed 100 pandemic-related allogeneic mobilized blood-derived stem cell apheresis products generated at 30 collection sites throughout Europe, shipped to and cryopreserved at our center between April and November of 2020. Products were shipped in the cool, subsequently frozen with DMSO as cryoprotectant. Irrespective of origin, all products were frozen within the prescribed shelf-life of 72 h.

**Results:**

Prior to cryopreservation, viable stem cell and leukocyte count according to the collection site and our reference laboratory were highly concordant (r^2^ = 0.96 and 0.93, respectively) and viability was > 90% in all instances. Median nominal post-thaw recovery of viable CD34+ cells was 42%. Weakly associated with poorer CD34+ cell recovery was higher leukocyte concentration, but not time lag between apheresis or addition of cryopreservant, respectively, and start of freezing. The correlation between pre- and post-thaw CD34+ cell dose was high (r^2^ = 0.85), hence predictable. Neutrophil and platelet engraftment were prompt with no evidence of dose dependency within the range of administered cell doses (1.31–15.56 × 10^6^ CD34+ cells/kg).

**Conclusions:**

General cryopreservation of allogeneic stem cell transplants is feasible. While more than half of the CD34+ cell content is lost, the remaining stem cells ensure timely engraftment.

**Supplementary Information:**

The online version contains supplementary material available at 10.1186/s12967-021-02810-9.

## Background

Techniques to maintain viability of cells in liquid nitrogen and revive them by thawing were developed in the 1970s; cryopreservation of “stem cells” is the backbone of autologous as well as cord blood transplantation. Allogeneic peripheral blood stem cell transplants, by comparison, are rarely subjected to freeze-thawing even though this might provide certain advantages: the logistics of transplant procurement and conditioning/transplantation can be disentangled, the risk of last-minute donor drop-out avoided. Transportation of fresh transplants to distant transplant centers can be challenging, given the short shelf life of fresh transplants, and non-cryopreserved transplants age from the moment they are collected into the bag. The ample experience with cryopreservation of mature cells, cord blood hematopoietic stem/progenitor cells (HSPCs) and autologous HSPCs as well as limited, sporadic experience with cryopreservation of allogeneic HSPCs strongly predicted feasibility of systematic cryopreservation of allogeneic transplants. On the other hand, freeze-thawing even under the best conditions damages cells, causing very significant loss of clonogenic activity of HSPC products [1] so that whether systematic cryopreservation would require adjustments to current apheresis practice, specifically to apheresis targets, seemed at least possible. The COVID-crisis now forced the community to involuntarily explore this issue. The transplant community was concerned about the need to defer donors or patients because of acute SARS-CoV-2 infection or quarantine, about redirection of medical resources to COVID-patients, away from apheresis and/or stem cell transplantation [2–4], about travel restrictions and, as a consequence thereof, about markedly reduced air travel options. Europe being a main region of origin of registry donor HSPC products as well as a major epicenter of the SARS-CoV-2 pandemic, the German central stem cell donor registry (ZKRD) accordingly opted to recommend cryopreservation of all MUD HSPC transplants. Specifically, the ZKRD requested to employ for all product slated for the Australian-Pacific region hub-and-spoke logistics, whereby aphereses were performed close to the donor’s place of residence, then shipped to a single central cryopreservation laboratory in Europe where they underwent processing and from where every couple of weeks the accumulated products were picked up and flown to Australia for distribution and shipping to the individual transplant centers. Some of the authors (EW, ES, HB) represent this central cryopreservation unit, German Red Cross Blood Service Baden-Württemberg-Hessen, Institute Frankfurt. A different strategy, not analyzed here, shipping of fresh HSPC product for cryopreservation at the transplant center, was selected by the NMDP for transplants slated for North American. The goal of the study is to describe the outcome of the hub-and-spoke logistic approach with respect to feasibility, especially assessment of the post-thaw pharmaceutical quality of allogeneic HSPC transplants with regards to cytometric and biological read-outs. The study further afforded the possibility to analyze systematic effects of certain variables – product age, cell density, etc. – on product quality. For this purpose, we analyzed 100 allogeneic HSPC products from 90 donors which were all cryopreserved at our institution because of the COVID-19 pandemic.

## Material and methods

### Apheresis, transportation and quality control

Registry donors were cleared, mobilized and apheresed per local donor site operating procedures and in accordance with national, European and WMDA guidelines. Products from 30 manufacturers throughout Europe were received. Product quality was locally assessed with respect to CD34+ cell (“HSPC”) viability, frequency, concentration, total dose and per-kg-dose. Some centers additionally declared T-cell and RBC dose and performed sterility testing. Products were hand-carried in temperature-controlled boxes equipped with temperature loggers at 4–8 °C and stored under those conditions until further handling. As soon as possible after receipt at our center, quality control samples were drawn by sterile-docking a small storage bag to assess HSPC viability, frequency and concentration (see viable CD34+ enumeration), leukocyte count (Sysmex 1800x, Norderstedt, Germany), T-cell frequency and concentration (Multi-test, BD) and MNC frequency (CD45/14, BD) as well as sterility (BacT/Alert, BioMérieux, Craponne, France [5]).

### Viable CD34+ enumeration

CD34+ concentration (ISHAGE HSPCs per µL), frequency (ISHAGE HSPCs among CD45+ events) and viability (frequency of 7-AAD-excluding ISHAGE HSPCs among total ISHAGE HSPCs) were assessed using the SCE kit (Becton–Dickinson, Heidelberg, Germany) as a single-platform. Briefly, 100 µL of fresh or thawed sample was stained with a pre-mixed combination of anti-CD34-PE (clone 8G12) and anti-CD45-FITC (clone 2D1) together with 7-AAD (vital dye) in TruCount Tubes (Becton–Dickinson). Samples were treated with non-fixating ammonium chloride solution without washing step (lyse-no-wash method). Samples were acquired on FACSCalibur (Becton–Dickinson) using stringent ISHAGE gating strategy. Detailed protocol and gating strategy were already published elsewhere [6].

### Cryopreservation

Cryopreservation in Germany underlies Good Manufacturing Practice (GMP) guidelines. This implies use of validated processes and qualified equipment throughout, and guidance by Standard Operating Procedures with meticulous documentation of manufacturing and quality control. Briefly, product was transferred to a centrifuge bag in a closed system using a graduated syringe which at the same time served to precisely measure product volume for calculation of total cell dose. WBC were pelleted by centrifugation. The target volume was calculated, aiming for a total WBC concentration no higher than 250,000/µL. Excess plasma beyond the target volume was discarded. Products with high WBC concentration were diluted with additional donor plasma provided with the product by the collection center where available, or with medicinal 20% human albumin (CSL Behring, Marburg, Germany). Plasma equivalent to half the target volume was transferred to a transfer bag, medicinal grade DMSO (Wak-Chemie, Steinbach/Taunus, Germany) was rapidly added to a concentration of 15% v/v (double-strength). DMSO-plasma was cooled on a cooling plate, subsequently transferred over 7 min to the double-strength cell suspension under constant light agitation of the cell suspension on a cooling plate. DMSO-containing cell suspension was distributed to labeled cryobags (Miltenyi Biotech, Bergisch Gladbach, Germany), 8 mL were retained for quality controls (WBC concentration, sterility) and retains. Cryobags were transferred into refrigerated aluminum cassettes. Three retains of 300 µL in cryogenic vials each were generated for each product. As quickly as possible but within no more than 45 min bags and tubes were moved to a controlled-rate freezer; until that time, products were held on refrigerated cooling pellets. The time between DMSO addition and cryopreservation start was determined using a stopwatch and documented on the batch record. The controlled-rate freezer (BV45, Consarctic, Schöllkrippen, Germany) cools the product at 1 K/min; at the eutectic point heating of the product is prevented by acutely cooling the chamber to − 45 °C. Once the product has reached − 40 °C, the cooling rate is increased to 5 K/min until − 100 °C have been reached at which point cassettes and retain tubes are transferred to liquid nitrogen vapor phase storage tanks. After a minimum of 72 h, one retain tube is thawed and leukocyte count (Sysmex) as well as WBC and HSPC viability (SCE kit) are measured. The specifications of the licensed medicinal product “HPC apheresis directed, cryopreserved, DRK-BSD Baden-Württemberg-Hessen (registration nr. PEI.G03648.01.2)” are listed in Additional file 1: Table S1.

### Data collection and statistics

Total dose, dose per kg and cell viability were extracted from product charts, WBC and CD34+ cell concentrations were calculated there from. Recovery was calculated as absolute number of viable cells in the post-thaw graft divided through absolute number of viable cells in fresh graft (in percent). Descriptive statistics were calculated in Excel for Office 2016 (Microsoft, Redmond, WA), linear regression analyses were performed and graphics generated in GraphPad Prism V.9 (San Diego, CA). Statistical significance of regressions is assumed at a p value < 0.05.

## Results

We analyzed a total of 100 allogeneic HSPC apheresis products collected at 30 European HSPC collection centers between April and November 2020 and shipped fresh to our cell processing center (CPC) for cryopreservation (Table 1). These 100 cryopreserved products were subsequently dispatched to 19 transplant centers worldwide (predominantly to Australia) for transplantation. For details on the HSPC products, see Table 2. Due to differences in national regulations, only CD34 dose and volume were consistently reported for all products received at the CPC. Reporting of other data necessary for clearance of stem cell products by our Qualified Person, such as T-cell dose or RBC volume, was variable and comparison thus not possible.

**Table 1:** Characteristics of HSPC apheresis products subjected to cryopreservation

	n	Median	Range (IQR)
Apheresis products	100		
Collection centers	30		
Apheresis products per collection center		2	1–19 (1–4.25)
Transplant centers	19		
Products per transplant center		3	1–27 (1–6)
Time between apheresis end and cryopreservation (h)	88	22	2.5–54 (21–24.00)
Time between DMSO addition and cryopreservation (min)	100	27	13–45 (21–33)
Product volume before cryopreservation (mL) – CPC	100	301	81–565 (222–351)
Product volume after apheresis (mL) – collection center	79	284	86–555 (224–358)

**Table 2:** Graft quality upon receipt and post-thaw

	n	Median	Range (IQR)
WBC			
Concentration (× 10^3^/µL)	100	189	102–581 (168–243)
Total cell number (× 10^10^)	100	5.74	1.29–16.25 (4.58–7.40)
Cell dose (× 10^6^/kg body weight)	100	817.4	165.9–5772 (582.3–1091)
Viability at receipt (%)	100	99.12	95.04–99.78 (98.89–99.39)
Viability after thaw (%)	100	63.11	41.60–83.66 (56.45–67.97)
Recovery viable WBC (%)	100	36	21–68 (30–41)
CD34+			
Concentration (cells/µL)	100	1649	246–10,345 (1200–2450)
Total cell number (× 10^6^)	100	484.5	39.6–1298 (323.5–675.6)
Cell dose (× 10^6^/kg body weight)	100	7.22	0.84–68.63 (4.78–9.51)
Viability at receipt (%)	100	99.61	92.33–100 (99.15–99.85)
Viability after thaw (%)	100	94.41	69.87–98.43 (90.77–95.94)
Recovery viable CD34+ (%)	100	42.20	23.20–92.40 (36.33–48.65)
Cell dose after thaw (× 10^6^/kg body weight)	100	2.81	0.39–29.60 (1.77–3.95)

Of the 90 donors (58 males and 32 females), ten (1 male and 9 females) were subjected to a second apheresis on the next day after failing to reach the requested CD34+ cell dose at day one. Donor demographics are depicted in Additional file 1: Table S2.

Since local policies in donor and apheresis management may affect the properties of the harvested product (Table 2) we analyzed the data collected from three countries in more detail. For all three countries at least 3 collection centers participated and at least total of 5 products were collected. The countries did not differ systematically in terms of WBC concentration in the apheresis product or shipping time (Additional file 1: Figure S1). HSPC doses from country one tracked requested doses more closely than from country two but both were very likely to meet the target dose with a single apheresis. Donors in the third country may be mobilized with less efficient regimes, and/or aphereses may not be run with optimal efficiency, leading to a 44% probability of failing to reach the minimum target of 4 × 10^6^ CD34+ cells/kg and necessitating subsequent second day apheresis. Despite the high frequency of second-day aphereses, the median total dose of HSPC per transplant (from one or two aphereses, as applicable) in country three failed to reach the typical minimal target in one third of cases.

**Fig. 1:**
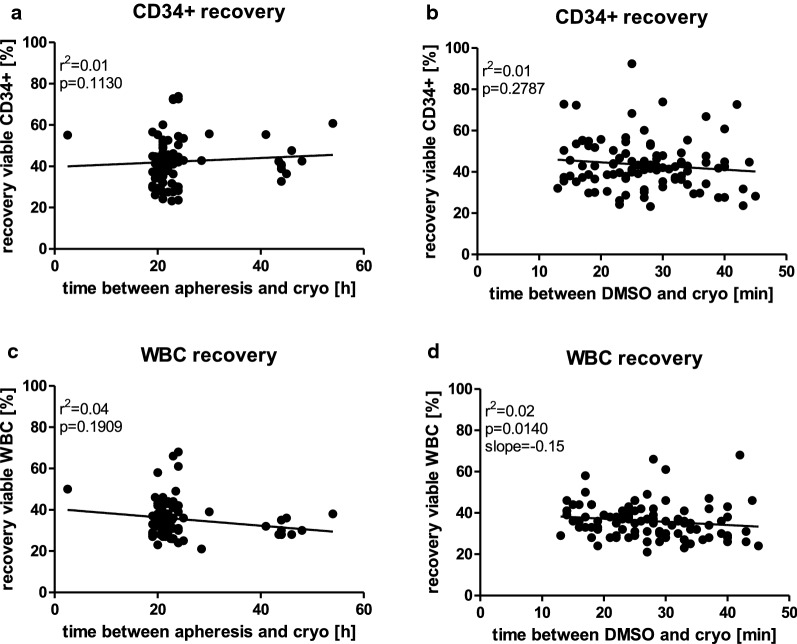
Effect of apheresis product age or duration of pre-freeze storage in DMSO-containing medium on post-thaw recovery. No correlation between post-thaw recovery and the age of the graft or the time lag between DMSO addition and start of cryopreservation. Each dot represents one apheresis product. Statistical measures are depicted in each graph

Next, we tested certain parameters which we hypothesized might impact CD34+ cell and WBC post-thaw recovery, namely transportation and holding time prior to cryopreservation (potential transportation damage) and time between addition of DMSO and initiation of the controlled freezing cycle (potential DMSO toxicity). Within the range of times covered by our observations, we neither observed transportation damage (range 2.5–54 h, Table 1), nor DMSO toxicity (range 13–45 min, Table 1) correlated with recovery of either WBC or CD34+ cells (Fig. 1a–d). Toxicity of DMSO is suggested to WBC (Fig. 1d) but not to CD34+ cells (Fig. 1c) by the observation of statistical significance (p = 0.0140) which is however negated by the fact that the slope of the calculated linear regression is not significantly different from 0. These observations support suitability of apheresis products for cryopreservation for at least 54 h and justify allowing up to 45 min for transfer of DMSO-replete apheresis product to the controlled rate freezer.

**Fig. 2:**
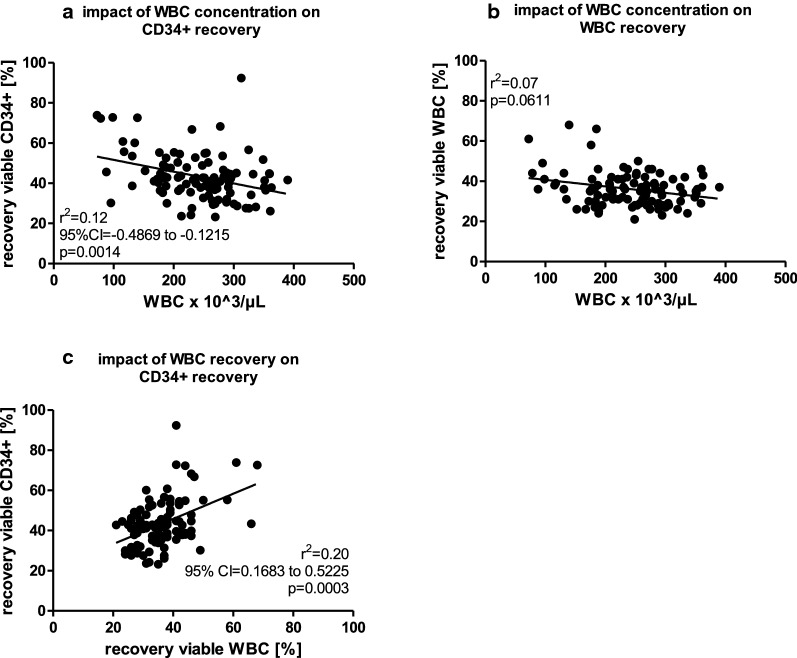
Post-thaw recovery as a function of apheresis product WBC concentration. Recovery of viable CD34+ cells correlates negatively but weakly with leukocyte concentration (**a**). WBC recovery is independent of its concentration (**b**). CD34+ recovery is associated with WBC recovery (**c**). Each dot represents one apheresis product. Statistical measures are depicted in each graph

In agreement with previously published data [7], higher WBC concentrations in the apheresis product were associated with below-average CD34+ cell recovery with no apparent threshold or pivot point within the range of our observations (Fig. 2a). A similar trend was observed for WBC recovery (Fig. 2b). Accordingly, a modest but statistically significant correlation between CD34+ and WBC recovery was observed (Fig. 2c).

**Fig. 3:**
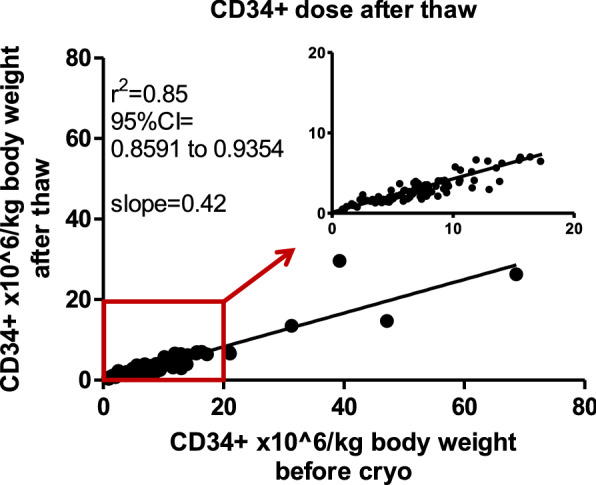
Correlation of pre- and post-thaw CD34+ dose. Post thaw CD34+ dose strongly correlates with CD34+ dose before cryopreservation. On average 42% of the CD34+ cells are recovered post-thaw, with a narrow margin of error. B is the magnified section of the graph in A. Each dot represents one apheresis product. Statistical measures are depicted in each graph

Unsurprisingly, CD34+ post thaw cell dose per kg recipient’s body weight strongly correlated with the initial dose before cryopreservation (r^2^ = 0.85, p < 0.0001, slope = 0.42) (Fig. 3). The strength of the correlation is sufficient to support its use for prediction of post-thaw dose recovery or apheresis target planning or, in other words, justifies use of pre-freeze quality data for clinical decision making as is current practice.

**Fig. 4:**
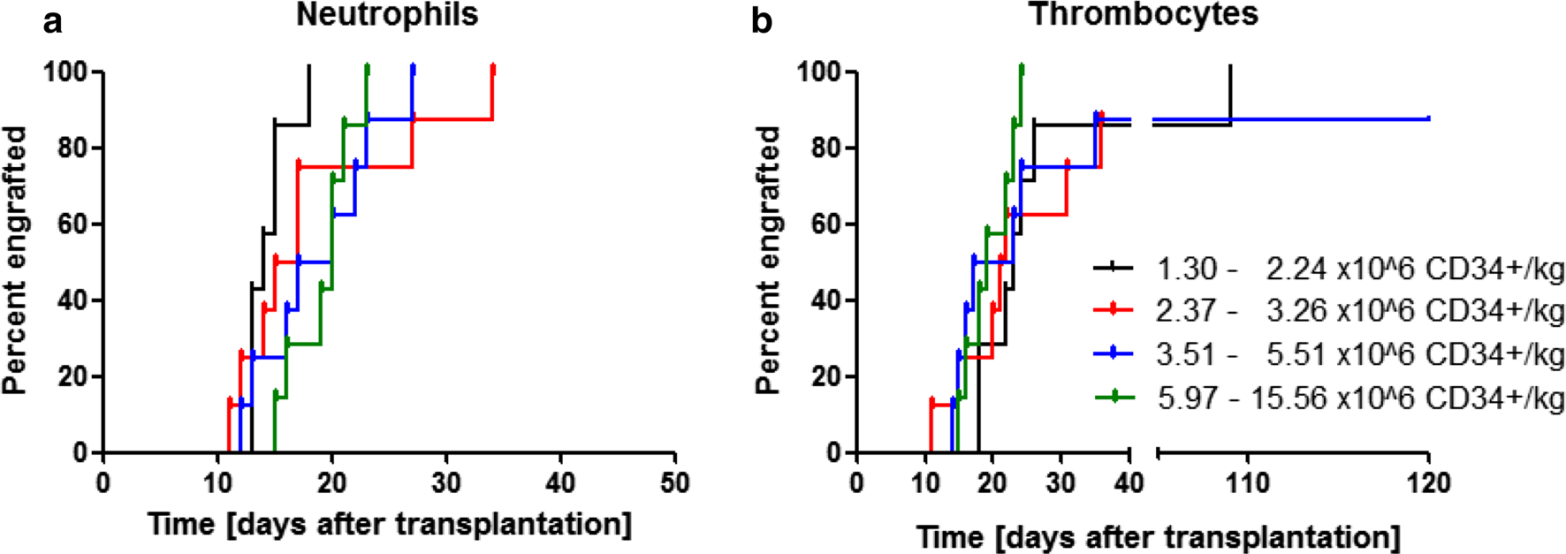
Engraftment kinetics as a function of CD34+ cell dose: Time to engraftment for neutrophils (**a**) or thrombocytes (**b**) was independent from the post-thaw dose of CD34+ cells. Neutrophil engraftment was defined as the first of three consecutive days with neutrophil counts > 500/µL, platelet engraftment as the first of three successive days with > 20,000/µL platelets without substitution

What constitutes a minimal safe therapeutic dose of CD34+ cells remains controversial. We sought to address whether the cryopreservation-inherent cell loss would result in delayed engraftment overall or begin to expose dose effects. Therefore, we evaluated the engraftment data from three local transplant centers which combined received one third of the products, i.e. 33 cryopreserved HSPC products for 30 patients (25 adults and 5 children/adolescents). With the exception of one child transplanted for a primary immunodeficiency, all transplant indications were refractory malignancies. 60% of the patients underwent myeloablative and 40% reduced intensity conditioning. Three of the patients received two HSPC products collected on consecutive days. The calculated median dose of the 30 thawed transplants was 3.38 × 10^6^ (range 1.31–15.56 × 10^6^; IQR 2.34–5.62 × 10^6^) CD34+ cells per kg recipient’s body weight. All patients engrafted with neutrophils, recovery being well within expectation with a median of 16.5 days (range 11–34 days; IQR 14–20 days) after transplantation (Fig. 4a and Additional file 1: Figure S2A). 93% (28 out of 30 patients) showed platelet engraftment at a median of 21.5 days (range 11–109 day; IQR 16–24 day) after transplantation (Fig. 4b and Additional file 1: Figure S2B). Two patients remained platelet transfusion dependent at the time of preparation of the manuscript on days+ 141 and+ 185 after transplantation (HSPC dose = 3.51 and 2.37 × 10^6^/kg respectively). One patient with severe acute GvHD engrafted with platelets as late as at day+ 109, the delay being presumably due to GvHD because neutrophil engraftment was very prompt (day 13; HSPC dose = 1.58 × 10^6^/kg). To identify potential dose effects, engraftment kinetics and probability were assessed by dose, whereby the patients were analyzed in quartiles according to calculated post-thaw CD34+ cell dose. As shown in Fig. 4, engraftment velocity was independent of post-thaw stem cell dose. Post-thaw recovery not being a specification, only the pre-freeze dose was disclosed to the transplant centers. Therefore, engraftment was similarly calculated using the pre-freeze dose; again a dose-dependency for engraftment velocity was not apparent within the range of observations (Additional file 1: Figure S3). We also queried whether products with above- or below-average recoveries would provide differential engraftment capacity; this was not the case (Additional file 1: Figure S4).

**Fig. 5:**
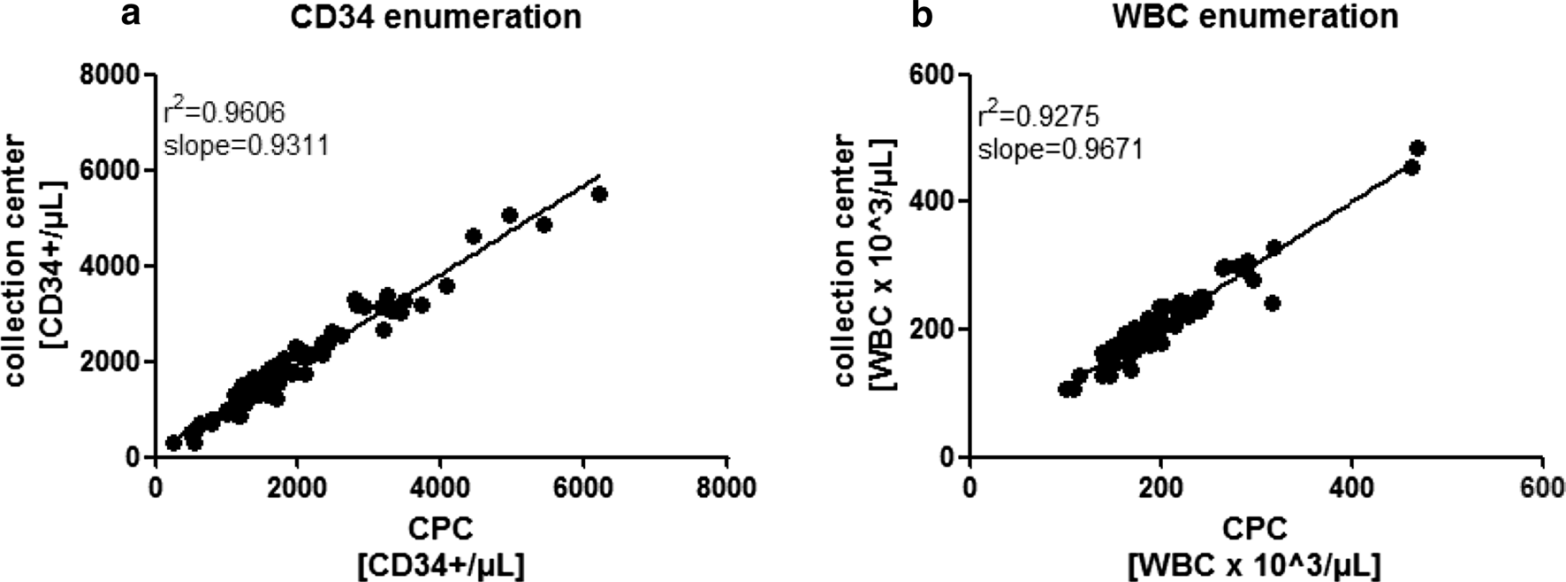
Correlation of CD34+ dose assessment in apheresis center and CPC: Nearly perfect accuracy and precision of CD34+ cell (**a**) and WBC (**b**) enumeration between 29 HSPC collection centers and the CPC indicates high robustness of current enumeration protocols. Incidentally, they also illustrate the high stability of the products over the observation time. Data from our own apheresis center were omitted for this analysis. Each dot represents one apheresis product. Statistical measures are depicted in each graph

Finally, the large number of external apheresis products from in total 29 external collection centers provided the unique opportunity to compare WBC and CD34+ enumeration results between collection centers and the CPC. We found nearly perfect precision and accuracy in both WBC and CD34+ cell enumeration (Fig. 5) indicating on the one hand the robustness of the ISHAGE HSPC enumeration protocol and, less remarkable, automated hemocytometry and on the other hand confirming the stability of the cell products within the time tested.

## Discussion

We observe that cryopreservation of registry donor apheresis products in a hub-and-spoke fashion is feasible. Product quality is well preserved over the entire duration of observation; the process is robust with respect to time lag between DMSO addition and freezing. Cell loss was significant, but its magnitude is foreseeable and, at least for pre-freeze CD34+ cell doses of not much below 4 × 10^6^/kg, irrelevant for transplant outcomes since even at the lowest transplant doses engraftment was timely.

The remarkable discrepancy between “viability”, i.e. the frequency of 7-AAD-excluding ISHAGE CD34+ cells among all ISHAGE CD34+ cells, and “recovery”, i.e. the fraction of recovered 7-AAD-excluding ISHAGE CD34+ cells relative to the pre-freeze dose begs discussion. Recovery being calculated from the post-thaw concentration of viable bona fide ISHAGE HSPCs in a pilot tube and the cryopreservation volume, it reflects the aggregate effects of loss due to quality control sampling, processing and freeze thawing and assumes comparable post-thaw quality in bags and companion tubes. Recently died cells, characterized by smaller size and higher side scatter, are excluded from the ISHAGE “viable cells” gate and less recently died cells move to the debris region. “Viability” thus markedly overestimates the residual dose of active substance (viable HSPCs) for that reason alone, as well as it does not account for cell loss during processing, e.g. centrifugation and quality control sampling. Direct comparison of the quality of our post-thaw recovery with what has been published is challenging, for several reasons: of the surprisingly limited number of published studies, the minority has looked at peripheral blood progenitor cells, even fewer at allogeneic peripheral blood progenitor cells. Moreover, the methods used to estimate cell recovery vary substantially between studies. Single-platform flow cytometric enumeration of absolute numbers of viable CD34+ post-thaw with direct reference to numbers obtained with the same method pre-cryo leads to more accurate results [8]. Thus, the average post-thaw recovery of CD34+ or colony forming cells vary somewhat between studies, but suggests recoveries between 40 and 70% [1, 8–14]. The recoveries reported here are, thus, in the range of the expected. We used a small pilot sample in a cryovial, not validated for this purpose, for the estimation of cell recovery, whereas, in as far as the sample type was at all reported, most of the others drew a sample directly from the infusion bag. The material of which the reservoir is made determines the heat conductivity during cryopreservation and thawing, with cryovials showing inferior characteristics to cryobags [15, 16]. Our automatic cryopreservation protocol is optimized for freezing of products in cryobags and not in cryovials. In conclusion, our calculations likely somewhat underestimate the true HSPC recovery in the infusion bag.

The most pertinent quality of a stem cell product is its ability to provide timely engraftment. For benchmarking, therefore, we analyzed engraftment data for our 75 allo-transplanted patients from 2018. All those 75 patients transplanted with fresh products achieved neutrophil engraftment. Median time to neutrophil engraftment was 18.0 days (95% CI 17.2–18.4). Platelet engraftment was achieved by 95% of patients, with a median time to engraftment for those that achieved engraftment of 19 days (95% CI 17.0–21.0). Hence, the comparison with data from 2018 underlines the very similar engraftment kinetics to what we here report for cryopreserved transplants. We show that median time to engraftment and probability of engraftment for neutrophils and platelets were maintained, and were both independent of the estimated post-thaw dose of infused viable CD34+ cells within the range of doses tested. As few as 1.31 × 10^6^ cells/kg resulted in timely engraftment. A threshold dose of stem cells for engraftment very likely exists but appears to be markedly lower than what is used clinically even under the worst of circumstances. This assumption is consistent with aggregate data from the late eighties and nineties on the engraftment performance of cryopreserved allogeneic grafts showing no correlation with cell dose [17]. A more recent analysis of historical data of cryopreserved allogenic transplants in the USA similarly suggested timely engraftment independent of the CD34+ recovery [18] and similarly concluded that cryopreservation did not put the recipient at risk. A recently reported Australian study of allogeneic grafts cryopreserved between 2015 and 2019 comes to the same conclusions re. engraftment, so it is unclear why the authors nevertheless raise concerns about the substantial loss of viable CD34+ cell counts post-thaw [7]. Kim et al. [1], performing CFU-C assays on residue from thawed HSPC product bags, report loss of 20–60% of clonogenic potential in thawed vs. fresh product, i.e. in an essentially similar range as what we are describing for loss of viable CD34+ cells, without an apparent effect thereof on engraftment kinetics. Adequate engraftment probability and kinetics are similarly suggested by the sizeable cohort analyzed by Parody and colleagues [19]. Very elegant engraftment data of cryopreserved grafts were recently put forth by Maurer et al. [20]. The rationale for cryopreservation was the same as in our series, COVID-19 response, and sensitive methods including T-cell chimerism were applied, in aggregate showing equivalent function of cryopreserved grafts compared to historic controls from the same center. One retrospective comparison of engraftment kinetics in 76 patients receiving cryopreserved grafts versus 123 recipients of fresh products shows significantly delayed platelet engraftment in the cryo group, albeit without significant impact on overall survival (although possibly underpowered for that parameter) [21]. Other risk factors, specifically much higher average donor and recipient age, may have contributed; neither factor was considered in the multivariate analysis. Multivariate analysis indeed identified lower pre-freeze CD34+ cell doses as correlated with lack of timely neutrophil or platelet engraftment; post-thaw recovery was not assessed. One other study showed an unexpectedly high incidence of graft failure in recipients of cryopreserved allogeneic HSPC products when compared to recipients of fresh graft [9]. The authors conclude that the outcome correlated with the ‘fitness’ of the stem cells measured by aldehyde dehydrogenase expression but not with the post-thaw viable CD34+ dose. However, post-thaw viable CD34+ cell numbers were not available or very low, i.e. below 2.0 × 10^6^ /kg of recipient body weight in 4 out of 6 patients who experienced engraftment failure. In conclusion, the majority of published data supports the safety of cryopreservation of allogeneic HSPC transplants and importantly also indicate that the typical collection target of 4–5 × 10^6^ CD34+ cells/kg body weight need not be adjusted to off-set the invariable loss from cryopreservation. In contrast to these data from allogeneic transplantation, previous studies on frozen autologous stem cell products had reported a relevant negative correlation between cell dose and engraftment time point [10, 22]. We propose that this observation is likely biased by patient variables, predominantly cumulative chemotherapy dose, impinging on stem cell quality [22, 23], not just quantity.

As we are showing, the ZKRD’s hub-and-spoke approach, with donor-proximal apheresis and central cryopreservation in Frankfurt, geographically located in the center of Europe and home to a major transportation hub with direct air traffic connections to all regions of the world, is feasible. The approach is likely preferable over fresh delivery of transplants to the transplant unit’s cell processing center and cryopreservation there, since time to freezing is shorter (although, as we are showing, aging effects are pleasantly mild), as well as bulk long-haul distance shipping of LN2 dewars as opposed to individual transportation of fresh products likely provides ecological and economic benefits. Clearly, thawed HSPCs are therapeutically active in that they provide reliable, even timely engraftment. However, due to donor safety reasons, indication for cryopreservation of an allogeneic stem cell product should be made according to strict guidelines because—as the COVID-crisis teaches—the drop-out rate of patients is considerable. At an interim analysis after the first wave of COVID had abated, the NMDP registry (National Marrow Donor Program, USA) reported up to 1% of not transfused products [24], whereas DKMS (Deutsche Knochenmarkspenderdatei, DE) on the other hand reported as many as 3.3% of cryopreserved products that definitively will not be transfused and another 7.4% of products with uncertain outcome [25]. DKMS estimates that the unnecessary collections associated with cryopreservation during COVID-19 pandemics will plateau at rates between 5 and 8% [25]. While unnecessary collections also occur with transplantation of fresh products, although the frequency is unknown, it is reasonable to assume that it is markedly lower.

Our analysis unexpectedly provided several other insightful pieces of information: Products are remarkably insensitive to storage time prior to cryopreservation as well as pre-freeze storage time in DMSO-containing media. Very high WBC concentrations are associated with inferior product quality and should likely be avoided. CD34+ cell enumeration is now extremely robust. Marked differences in apheresis outcomes between countries suggest potential benefit of additional measures of standardization of best-practice approaches with respect to donor mobilization and apheresis management.

Limitations of the work must be acknowledged. The claim of feasibility of hub-and-spoke logistics for stem cell procurement is based for now on a single example, ourselves. Ecological and economic analyses could enhance the discussion. The pharmacologically active substance, viable HSPCs, was enumerated immunophenotypically, although post-thaw this may over-estimate potency. Engraftment supersedes for in vitro clonogenic assays, but the analyzed patient cohort for engraftment is small, the analysis limited to myeloid engraftment, and the long-term effects after transplantation are not yet studied. Moreover, functional assays and/or profound analysis of stem cell subsets, although not feasible in a daily clinical routine, could possibly better characterize graft properties. In addition, the quality of the retain samples—thick-walled cryo vials, low-volume samples—may systematically underestimate stem cell recovery.

## Conclusions

In summary, regionally centralized cryopreservation of registry donor apheresis products is not only feasible and yields favorable transplant outcomes, it may also be economically and ecologically preferable as well as beneficial for health care resource management. The practice could outlive the COVID-19 crisis if transplant centers manage to improve their ability to identify patients who will lose transplant eligibility until their scheduled transplantation date.

## Supplementary Information

**Additional file 1.** Product specifications, donor demographics, as well as some additional detailed data analyses are displayed.

## Data Availability

Not applicable.
