# Supplementary material for: Allogeneic transplant procurement in the times of COVID-19: Quality report from the central European cryopreservation site
Source: J Transl Med. 2021 Apr 8;19:145. doi: 10.1186/s12967-021-02810-9 (PMC8027980; doi:10.1186/s12967-021-02810-9)
Supplement: Supplementary file 1 — Additional file 1. Product specifications, donor demographics, as well as some additional detailed data analyses are displayed. [file 12967_2021_2810_MOESM1_ESM.pptx]

## Slide 1
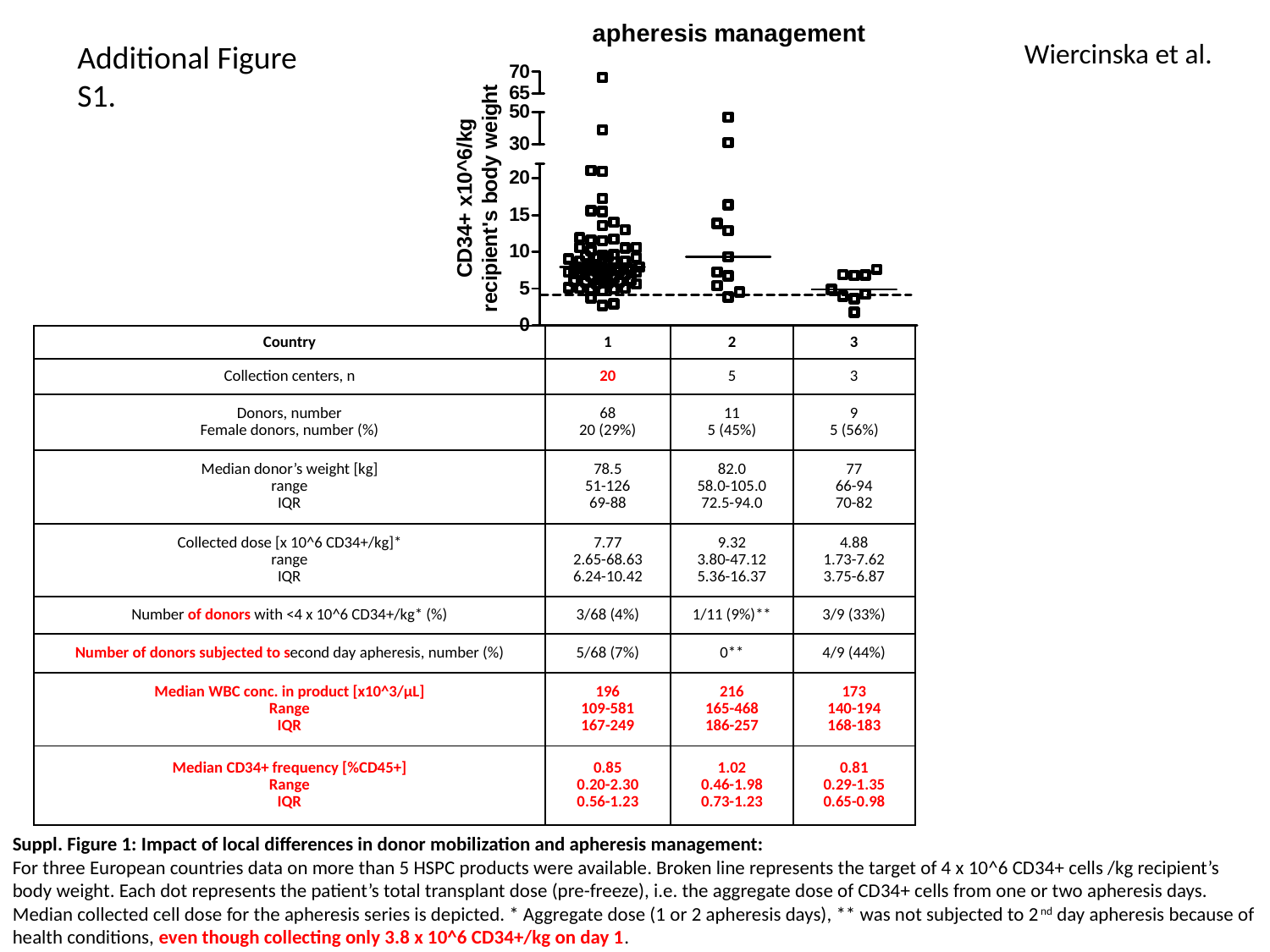

Wiercinska et al.
Additional Figure S1.
| Country | 1 | 2 | 3 |
| --- | --- | --- | --- |
| Collection centers, n | 20 | 5 | 3 |
| Donors, number Female donors, number (%) | 68 20 (29%) | 11 5 (45%) | 9 5 (56%) |
| Median donor’s weight [kg] range IQR | 78.5 51-126 69-88 | 82.0 58.0-105.0 72.5-94.0 | 77 66-94 70-82 |
| Collected dose [x 10^6 CD34+/kg]\* range IQR | 7.77 2.65-68.63 6.24-10.42 | 9.32 3.80-47.12 5.36-16.37 | 4.88 1.73-7.62 3.75-6.87 |
| Number of donors with <4 x 10^6 CD34+/kg\* (%) | 3/68 (4%) | 1/11 (9%)\*\* | 3/9 (33%) |
| Number of donors subjected to second day apheresis, number (%) | 5/68 (7%) | 0\*\* | 4/9 (44%) |
| Median WBC conc. in product [x10^3/µL] Range IQR | 196 109-581 167-249 | 216 165-468 186-257 | 173 140-194 168-183 |
| Median CD34+ frequency [%CD45+] Range IQR | 0.85 0.20-2.30 0.56-1.23 | 1.02 0.46-1.98 0.73-1.23 | 0.81 0.29-1.35 0.65-0.98 |
Suppl. Figure 1: Impact of local differences in donor mobilization and apheresis management:
For three European countries data on more than 5 HSPC products were available. Broken line represents the target of 4 x 10^6 CD34+ cells /kg recipient’s body weight. Each dot represents the patient’s total transplant dose (pre-freeze), i.e. the aggregate dose of CD34+ cells from one or two apheresis days. Median collected cell dose for the apheresis series is depicted. * Aggregate dose (1 or 2 apheresis days), ** was not subjected to 2nd day apheresis because of health conditions, even though collecting only 3.8 x 10^6 CD34+/kg on day 1.

## Slide 2
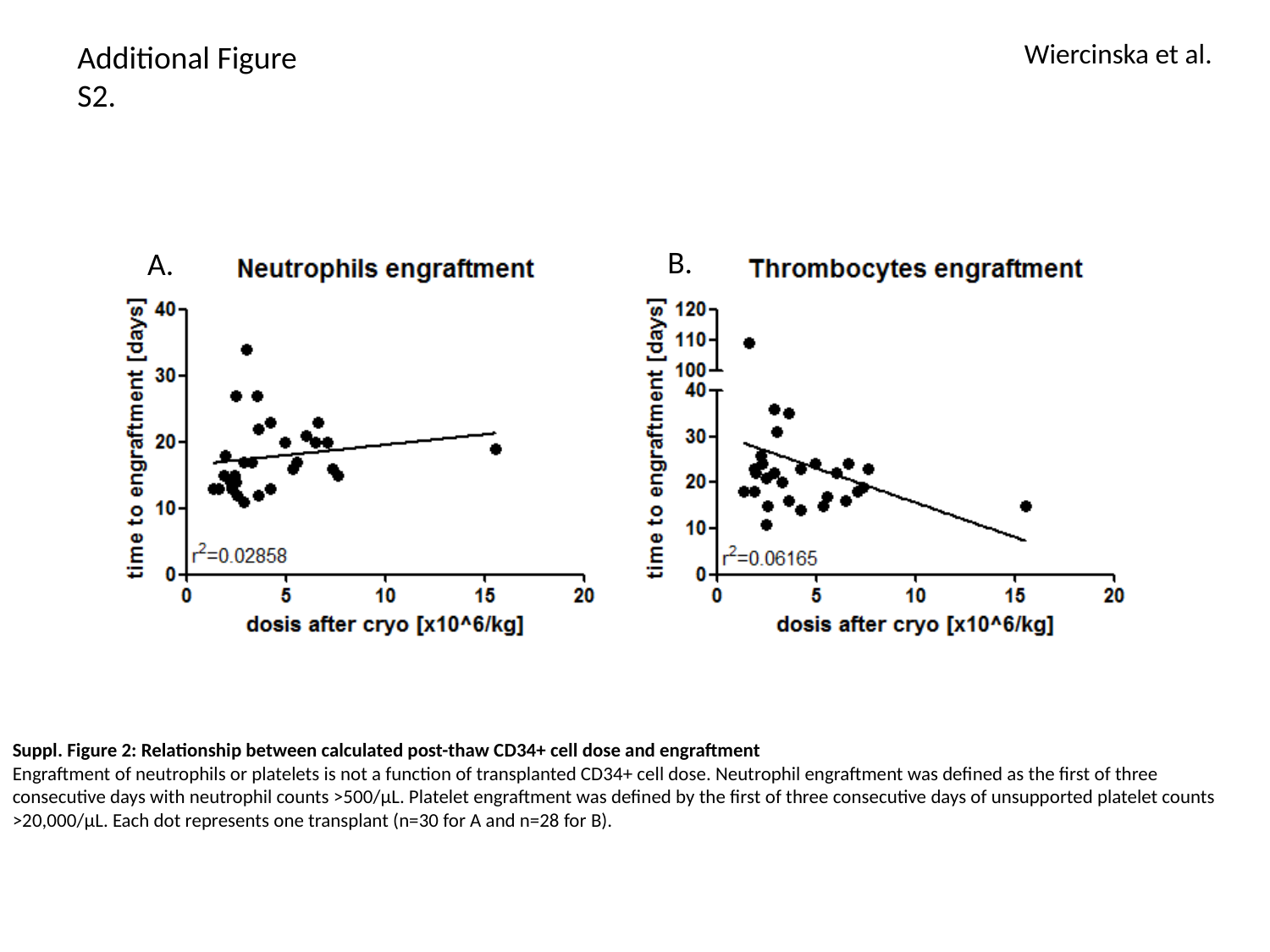

Wiercinska et al.
Additional Figure S2.
B.
A.
Suppl. Figure 2: Relationship between calculated post-thaw CD34+ cell dose and engraftment
Engraftment of neutrophils or platelets is not a function of transplanted CD34+ cell dose. Neutrophil engraftment was defined as the first of three consecutive days with neutrophil counts >500/µL. Platelet engraftment was defined by the first of three consecutive days of unsupported platelet counts >20,000/µL. Each dot represents one transplant (n=30 for A and n=28 for B).

## Slide 3
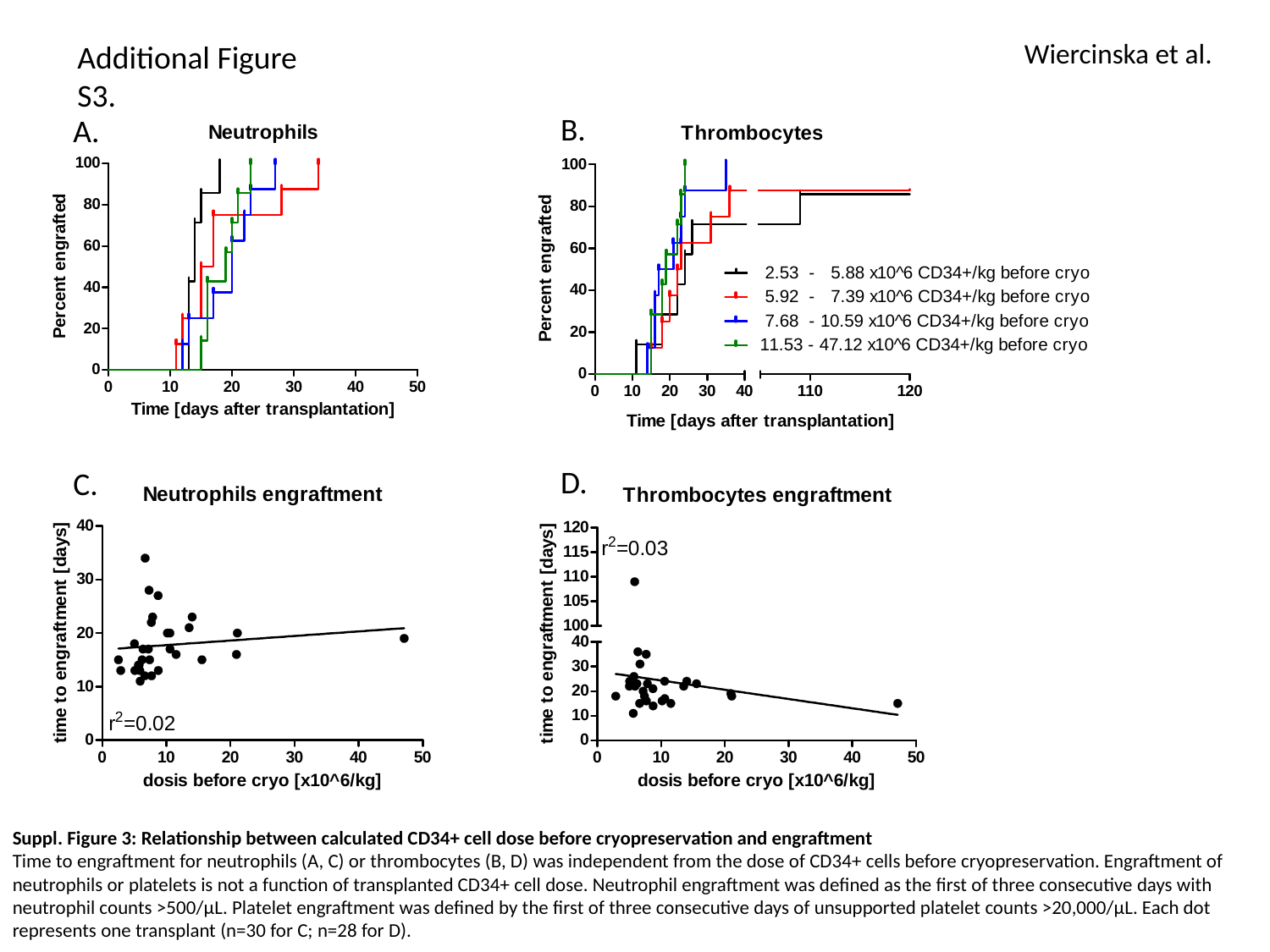

Wiercinska et al.
Additional Figure S3.
B.
A.
D.
C.
Suppl. Figure 3: Relationship between calculated CD34+ cell dose before cryopreservation and engraftment
Time to engraftment for neutrophils (A, C) or thrombocytes (B, D) was independent from the dose of CD34+ cells before cryopreservation. Engraftment of neutrophils or platelets is not a function of transplanted CD34+ cell dose. Neutrophil engraftment was defined as the first of three consecutive days with neutrophil counts >500/µL. Platelet engraftment was defined by the first of three consecutive days of unsupported platelet counts >20,000/µL. Each dot represents one transplant (n=30 for C; n=28 for D).

## Slide 4
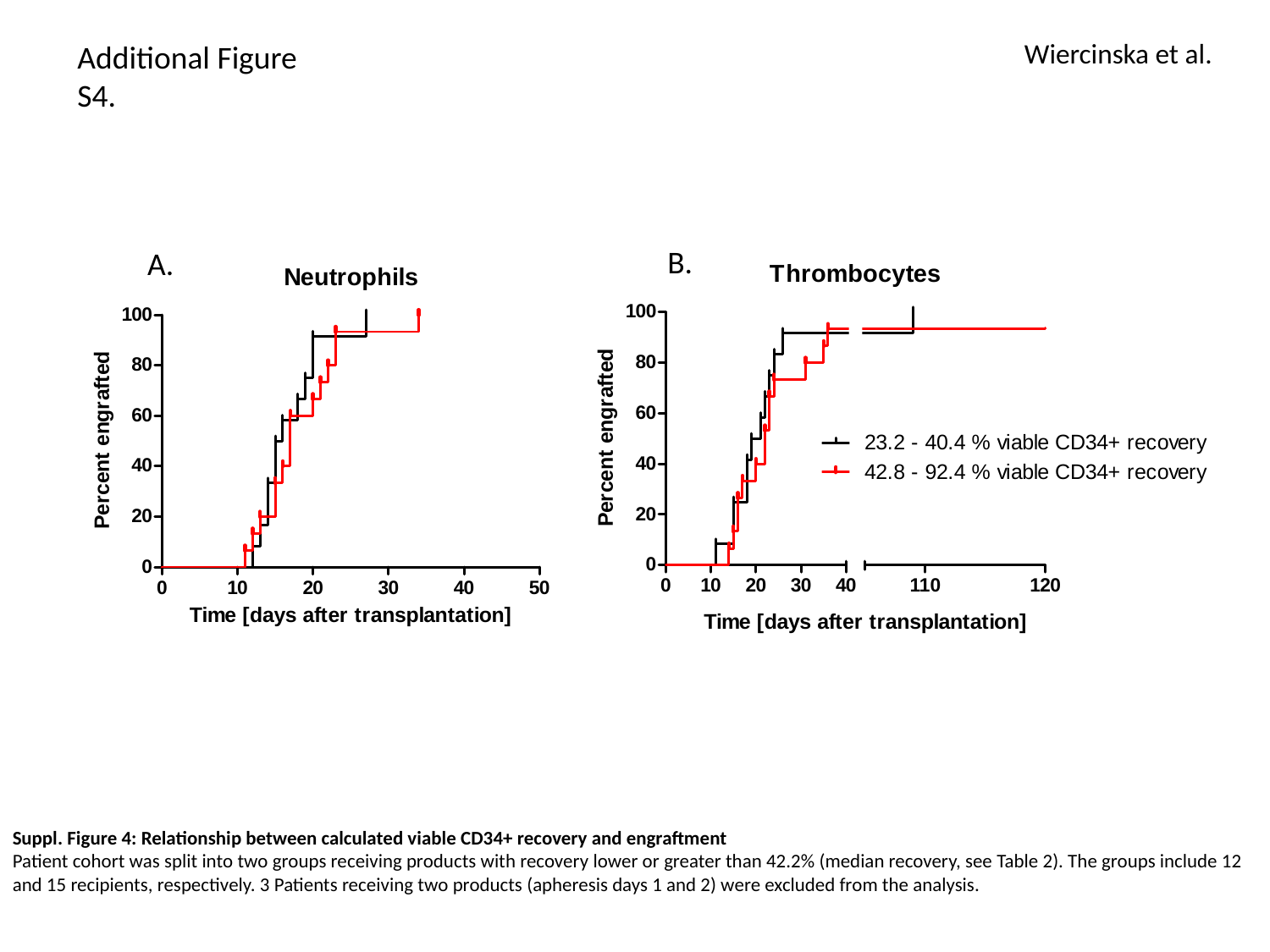

Wiercinska et al.
Additional Figure S4.
B.
A.
Suppl. Figure 4: Relationship between calculated viable CD34+ recovery and engraftment
Patient cohort was split into two groups receiving products with recovery lower or greater than 42.2% (median recovery, see Table 2). The groups include 12 and 15 recipients, respectively. 3 Patients receiving two products (apheresis days 1 and 2) were excluded from the analysis.

## Slide 5
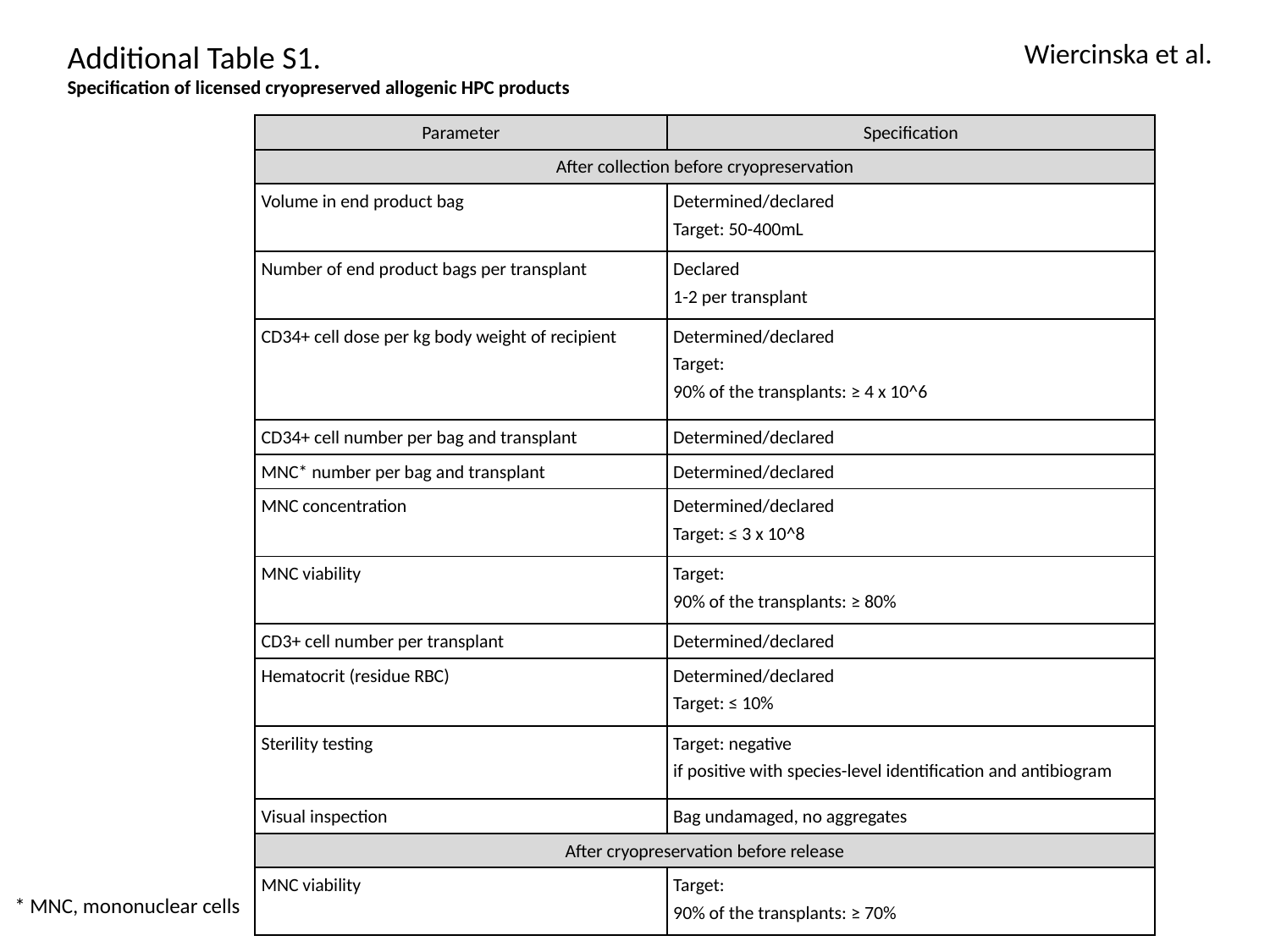

Wiercinska et al.
Additional Table S1.
Specification of licensed cryopreserved allogenic HPC products
| Parameter | Specification |
| --- | --- |
| After collection before cryopreservation | |
| Volume in end product bag | Determined/declared Target: 50-400mL |
| Number of end product bags per transplant | Declared 1-2 per transplant |
| CD34+ cell dose per kg body weight of recipient | Determined/declared Target: 90% of the transplants: ≥ 4 x 10^6 |
| CD34+ cell number per bag and transplant | Determined/declared |
| MNC\* number per bag and transplant | Determined/declared |
| MNC concentration | Determined/declared Target: ≤ 3 x 10^8 |
| MNC viability | Target: 90% of the transplants: ≥ 80% |
| CD3+ cell number per transplant | Determined/declared |
| Hematocrit (residue RBC) | Determined/declared Target: ≤ 10% |
| Sterility testing | Target: negative if positive with species-level identification and antibiogram |
| Visual inspection | Bag undamaged, no aggregates |
| After cryopreservation before release | |
| MNC viability | Target: 90% of the transplants: ≥ 70% |
* MNC, mononuclear cells

## Slide 6
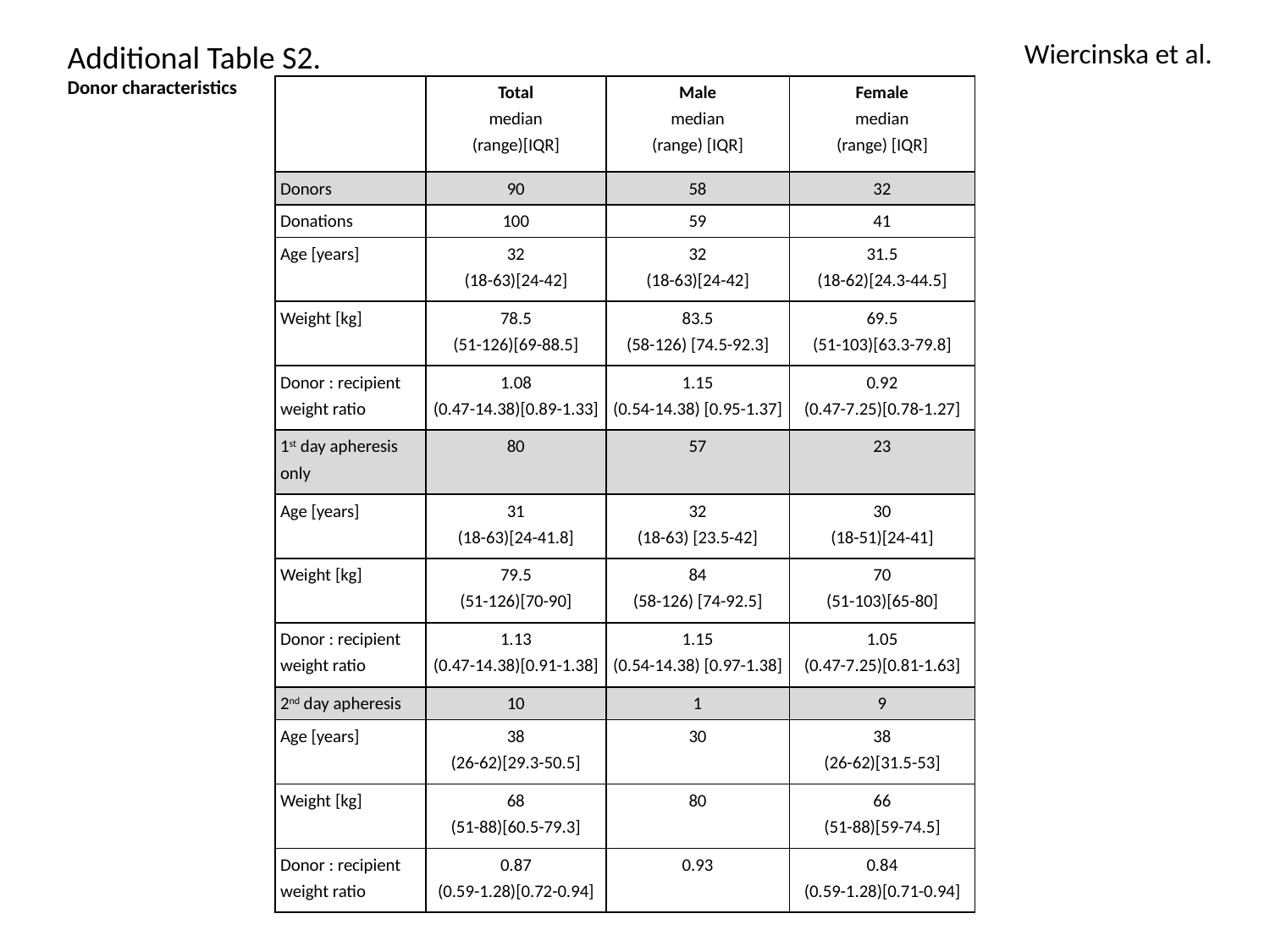

Wiercinska et al.
Additional Table S2.
Donor characteristics
| | Total median (range)[IQR] | Male median (range) [IQR] | Female median (range) [IQR] |
| --- | --- | --- | --- |
| Donors | 90 | 58 | 32 |
| Donations | 100 | 59 | 41 |
| Age [years] | 32 (18-63)[24-42] | 32 (18-63)[24-42] | 31.5 (18-62)[24.3-44.5] |
| Weight [kg] | 78.5 (51-126)[69-88.5] | 83.5 (58-126) [74.5-92.3] | 69.5 (51-103)[63.3-79.8] |
| Donor : recipient weight ratio | 1.08 (0.47-14.38)[0.89-1.33] | 1.15 (0.54-14.38) [0.95-1.37] | 0.92 (0.47-7.25)[0.78-1.27] |
| 1st day apheresis only | 80 | 57 | 23 |
| Age [years] | 31 (18-63)[24-41.8] | 32 (18-63) [23.5-42] | 30 (18-51)[24-41] |
| Weight [kg] | 79.5 (51-126)[70-90] | 84 (58-126) [74-92.5] | 70 (51-103)[65-80] |
| Donor : recipient weight ratio | 1.13 (0.47-14.38)[0.91-1.38] | 1.15 (0.54-14.38) [0.97-1.38] | 1.05 (0.47-7.25)[0.81-1.63] |
| 2nd day apheresis | 10 | 1 | 9 |
| Age [years] | 38 (26-62)[29.3-50.5] | 30 | 38 (26-62)[31.5-53] |
| Weight [kg] | 68 (51-88)[60.5-79.3] | 80 | 66 (51-88)[59-74.5] |
| Donor : recipient weight ratio | 0.87 (0.59-1.28)[0.72-0.94] | 0.93 | 0.84 (0.59-1.28)[0.71-0.94] |
